# Non-diabetic ketoacidosis associated with a low carbohydrate, high fat diet in a postpartum lactating female

**DOI:** 10.1093/omcr/omz026

**Published:** 2020-08-10

**Authors:** Saba Marzban, Mohamed Arbee, Naseema Vorajee, Guy A Richards

**Affiliations:** 1 Clinical Pharmacy, Netcare Christiaan Barnard Memorial Hospital, Cape Town, South Africa; 2 Clinical Haematology, Netcare Garden City Hospital, Johannesburg, Gauteng, South Africa; 3 Division of Pathology, National Institute for Occupational Health, National Health Laboratory Services, Johannesburg, Gauteng, South Africa; 4 Division of Critical Care Charlotte Maxeke Hospital and University of the Witwatersrand, Faculty of Health Sciences

**Keywords:** LCHF, lactation, ketoacidosis

## Abstract

A 31-year old non-diabetic woman presented to our hospital with symptoms of dehydration, drowsiness, fatigue, shortness of breath and vomiting present for two consecutive days prior to admission. She had started a low carbohydrate, high fat (LCHF) diet to induce weight loss while breastfeeding her 4-month-old child 2 weeks prior to admission. The patient was found to have a severely high anion gap metabolic acidosis. It was determined to be due to ketoacidosis, which was as a result of carbohydrate restriction in the presence of increased metabolic demands related with the synthesis and secretion of milk. She denied alcohol use or ingestion of any drugs prior to admission. The patient underwent dialysis and received insulin, 5% dextrose water alongside a well-balanced diet with adequate calories. All abnormal laboratory results normalized and follow-up visits were done. Lactating women are at risk of developing ketoacidosis due to high metabolic demands of the body to produce milk. LCHF diets may exacerbate the body’s demand to meet its milk production requirement and result in ketoacidosis. Health professionals need to be aware of the complications of LCHF diet in this population to prevent mortality associated with this condition.

## INTRODUCTION

Ketoacidosis is a type of metabolic acidosis with an elevated anion gap that can occur in starvation, uncontrolled diabetes, alcohol consumption and drug ingestion [1, 2]. The low glucagon:insulin ratio due to low insulin production and glycogen depletion as well as the ongoing caloric depletion from breastfeeding results in an increased fatty acid oxidation to generate adenosine triphosphate for energy consumption. This is a characteristic of lactation or bovine ketoacidosis. It results in an increased production of ketone bodies (acetoacetate, β-hydroxybutyrate and acetone) by the liver (ketosis), which results in a metabolic acidosis due to the increase in anions [3, 4, 6, 8].

Ketoacidosis during lactation in non-diabetic women is a rare event and to the best of our knowledge there has been nine case reports previously reported similar to our patient [1–9]. It is, however, seen commonly in chronically lactating cows and is a well-known concept in veterinary medicine. The increased metabolic demand from the production and secretion of milk exceeds the amount of carbohydrates ingested. This condition is, however, rare in lactating humans, as a balanced diet satisfies additional nutritional requirements [3, 10]. A diet low in carbohydrates and high in fat content mimics the metabolic changes induced by starvation where stored fat is utilized for energy consumption. This is particularly prevalent where insulin levels are low and glycogen stores are depleted as a result of glycogenolysis. Ketone bodies become the primary source of energy that is evident in elevated levels of ketones in serum and urine [11, 12]. Urine levels only represent a fraction of the serum levels and hence measurement of ketonaemia is a more accurate method of assessing the severity of ketoacidosis.

The low carbohydrate, high fat (LCHF) diet became popular in the 1920s as a method of treating type 1 diabetes as well as uncontrollable seizures in epileptic children before more effective antiepileptic medications were discovered [13]. It again became popular in the USA during the 1960s as a dietary method after the books of Stillman and Atkins [14, 15] were published. In South Africa the LCHF diet, also referred to as Banting, was popularized in 2013 by a South African Professor of Exercise and Sports Science at the University of Cape Town, Tim Noakes, a charismatic figure who garnered immense interest among the public.

## CASE REPORT

A 31-year old 4-month postpartum female, weighing 75 kg, presented to the emergency department with a history of drowsiness, fatigue, shortness of breath, vomiting and progressive confusion for two consecutive days prior to admission. She had initiated the LCHF diet about 2 weeks prior to admission while breastfeeding, in order to lose weight accumulated during pregnancy. She successfully lost 5 kg in 2 weeks following severe restriction of dietary carbohydrates. She denied any alcohol or drug use. She reported no known allergies or chronic conditions (including diabetes) except for mild asthma.

Her initial examination in the emergency department revealed a blood pressure of 133/89 mmHg, a heart rate of 126 beats/min, a respiratory rate of 30 breaths/min, oxygen saturation of 100% on room air, a temperature of 36.2°C and a haemoglucotest of 6.7 mmol/L. The pH on the arterial blood gases was 7.128; the pCO2 was 6.7 mmHg and the pO2 was 119 mmHg. Her lactate was 2.4 mmol/L (reference range 0.5–1.2 mmol/L) and glucose 9.1 mmol/L. Her urine dipstick chemistry revealed a pH of 5.5, ketones 4+ positive, blood 3+ positive and protein 1+ positive. Physical examination of her abdomen and chest were normal. Apart from her confusion, her central nervous system examination was normal with no localizing signs. Her blood results are explained in Table 1.

**Table 1:** Blood results

Blood results	Measured results	Reference range
Glycated haemoglobin	5.0	4.0–6.0
Morning cortisol	>1600 nmol/L	185–624 nmol/L
Urea	8 .7 mmol/L	
Creatinine	134 mmol/L	
Estimated glomerular filtration rate	46 ml/min	>90 ml/min

These results confirmed a non-diabetic ketoacidosis with acute kidney injury. Serum toxicology (benzodiazepines, barbiturates, paracetamol, salicylate, tricyclic antidepressants) results were negative.

She was admitted to the adult intensive care unit and received haemodialysis at the speed of QB 200 ml/min and QD 500 ml/min for 4 h. A high potassium bath was added as the serum potassium was found to be low [2.5 mmol/L (reference range 3.5–5.1 mmol/L)]. An insulin infusion was started at 5 units/h with a total of 45 units administered intravenously. This was followed by 6 units of insulin subcutaneously. Simultaneously, 5% dextrose water with 40 mmol potassium chloride was administered at 160 ml/h for 9 h, reduced to 100 ml/h for 5 h and then further reduced to 80 ml/h. Her acidosis was reversed after 24 h.

Empiric antibiotic therapy was administered to cover the possibility of sepsis although the CRP was only marginally elevated [22 mg/l (reference range >5 mg/l)], with a white blood cell count of 26.92 10^9/l (reference range 3.92–9.88 10^9/l), with neutrophils comprising 23.85 10^9/l (reference range 2.00–7.50 10^9/l) and monocytes 1.72 10^9/l (reference range 0.18–1.00 10^9/l) of the total cells.

Thyroid ultrasound showed increased vascularity of the thyroid gland. Levothyroxine 100 mcg twice daily was administered orally while she was admitted. Her thyroid hormone levels are depicted in Table 2.

**Table 2:** Thyroid and lipogram results

	Measured	Reference range
Thyroid stimulating hormone (TSH)	56.71 mIU/L	0.27–4.20 mIU/L
Free thyroxine (T4)	5 pmol/L	12–22 pmol/L
Triiodothyronine (T3)	1.5 pmol/L	3.1–6.8 pmol/
Antithyroglobulin	619 IU/ml	<116 IU/ml
Antithyroid peroxidase	>1000 IU/ml	<10 IU/ml

The amylase and lipase 48 h after admission were elevated at 98 U/L (reference range <100 U/L) and 299 U/L (reference range 13–60 U/L), respectively. These decreased during her admission. A sonar exam of the abdomen and pelvis showed no abnormalities. A chest X-ray and brain CT scan revealed no abnormalities as did the cardiac echo.

The lipogram profile (as shown in Table 3) was measured 2 days after admission and showed an elevated LDL cholesterol and non-HDL cholesterol both of which normalized 48 h after admission without pharmacological treatment. The triglyceride levels were also slightly elevated (still within the normal range) and decreased to 1.0 mmol/L on discharge.

**Table 3:** Lipogram profile

	Measured	Reference range
Low density lipoprotein (LDL) cholesterol	4.0 mmol/L	1.5–2.9 mmol/L
High density lipoprotein (HDL) cholesterol	1.8 mmol/L	1.2–1.9 mmol/L
Non-HDL cholesterol	5.1 mmol/L	0.9–3.7 mmol/L
Triglyceride	1.4 mmol/L	0.4–1.6 mmol/L

The patient recovered fully and was discharged after 4 days. She received a prescription for levothyroxine 100 mcg once daily orally but without insulin or oral hypoglycemics. Four follow-up visits post discharge showed normalization of amylase and lipase levels and thyroid and kidney function.

## DISCUSSION

Decreasing carbohydrates in the diet does induce weight loss, but no more than other named diets that are strictly adhered to [16]. There are, however, numerous potential ill effects of the LCHF, Banting diet. The most frequent of these is gout that is related to the increased protein intake in place of carbohydrates and the metabolic acidosis that results from extreme restriction of carbohydrates or from increased metabolic demand [17]. Metabolic ketoacidosis as a result of starvation is due to a decline in insulin levels leading to mobilization and incomplete oxidation of free fatty acids with a resultant increase in the concentration of the ketone bodies, acetoacetate and β-hydroxybutyrate. Acetoacetate is further decarboxylated to yield acetone [18], which is mainly excreted as volatile gas in the lungs [19]. Elevated levels result in metabolic acidosis that can be fatal due to secondary mitochondrial and endothelial dysfunction. It is essential therefore for health professionals to be aware of the signs and symptoms of this condition [20].

According to a study done by E. Kose *et al*., the LCHF diet mimics starvation and as such `down regulates the hypothalamic–pituitary–thyroid axis’. This subsequently inhibits anabolism and reduces the production of T3 from T4 [21]. Our patient had elevated levels of TSH, lowered levels of T3 and T4 and positive thyroid antibody levels, suggestive of Hashimoto’s disease. It is postulated that the LCHF diet may have unmasked the myxoedema. Therapy, as with diabetic ketoacidosis, requires replacement of glucose and insulin to convert the energy source to carbohydrate. Whether dialysis was necessary and contributed to the short recovery is debatable as it was initiated before a definitive diagnosis was made.

In summary, the metabolic demands of lactation may exceed that provided by carbohydrate consumption, glycogen stores and gluconeogenesis especially when carbohydrate consumption is severely reduced necessitating the use of fat for energy. This can lead to a potentially fatal ketoacidosis. We thus recommend that those at risk of ketoacidosis refrain from using the LCHF, Banting diet.

## CONFLICT OF INTEREST STATEMENT

None declared.

## ETHICS APPROVAL

Approval for publication was received from Netcare research operations committee. A copy of the approval is available for review by the editor-in-chief of this journal.

## CONSENT

Written informed consent was obtained from the patient for publication of this case report. A copy of the written consent is available for review by the editor-in-chief of this journal.

## AUTHORS’ CONTRIBUTIONS

All authors have been involved in all stages of the preparation of this case report and they have all read and approved the final version of this report.
